# Sequential EMDR and CBT for Small‐t Trauma in an Adult With Autism Spectrum Disorder: A Clinical Case Report

**DOI:** 10.1155/crps/4899601

**Published:** 2026-10-22

**Authors:** Keita Kawai, Michie Ishida, Yutaka Omura

**Affiliations:** ^1^ Department of Psychiatry and Psychotherapy Medical Center — University of Freiburg Faculty of Medicine University of Freiburg Freiburg Germany uni-freiburg.de; ^2^ Heian-dori Clinic Nagoya Aichi Japan

**Keywords:** autism spectrum disorder, case report, cognitive behavioral therapy, eye movement desensitization and reprocessing, small-t trauma

## Abstract

This clinical case report describes a sequential combination of eye movement desensitization and reprocessing (EMDR) therapy and cognitive behavioral therapy (CBT) for small‐t trauma in an adult patient with autism spectrum disorder (ASD). Individuals with ASD could be vulnerable to the accumulation of nonlife‐threatening adverse interpersonal experiences, often described as small‐t trauma, which can contribute to anxiety, depressive symptoms, and social withdrawal. The patient was a 25‐year‐old woman with ASD who presented with flashback‐like symptoms and social anxiety related to distressing childhood and adolescent experiences with family members and school‐related experiences. Treatment consisted of EMDR sessions targeting these traumatic memories, followed by CBT focused on cognitive restructuring and adaptive interpersonal coping. Across treatment, traumatic stress, anxiety, and depressive symptoms decreased, and the patient transitioned from unemployment to employment through a disability employment scheme. This case illustrates how EMDR may reduce trauma‐related distress sufficiently to allow subsequent CBT work on current interpersonal difficulties. Although causal conclusions cannot be drawn from a single case, this report suggests that EMDR adapted to the characteristics of ASD, followed by CBT, may be clinically useful for addressing small‐t trauma in adults with ASD.

## 1. Introduction

Autism spectrum disorder (ASD) is a neurodevelopmental disorder characterized by persistent deficits in social communication and social interaction across multiple contexts, as well as restricted, repetitive patterns of behavior, interests, or activities [1]. Symptoms emerge in the early developmental period and cause clinically significant impairment in functioning. ASD is a common neurodevelopmental disorder, with an estimated global prevalence of ~1%–2.8% [2, 3]. Individuals with ASD tend to develop other psychiatric disorders, and more than 70% of individuals present with at least one additional comorbidity and have higher rates of suicidal ideation and self‐injury behavior compared to the general population [4, 5]. In particular, individuals with ASD who receive a diagnosis in later life are more likely to have psychiatric comorbidities than those diagnosed in childhood, partly because years of unrecognized ASD often require sustained camouflaging of their difficulties [6, 7]. Since the underlying mechanisms remain unclear, it has been suggested that the ASD core symptoms themselves could be the risk factors for the onset of other psychiatric disorders. For example, one study revealed that the loneliness resulting from autism features could be linked to higher levels of depression and social anxiety [8]. Another study showed that emotional regulation and intolerance of uncertainty were associated with depression and anxiety in ASD [9]. Furthermore, several barriers have been identified that prevent individuals with ASD from seeking mental health treatment. Specifically, these individuals are often reluctant to seek support due to social stigma and a lack of knowledge, in addition to a shortage of clinicians specializing in adult ASD patients [10, 11].

Posttraumatic stress disorder (PTSD) is a prevalent psychiatric disorder, with a global prevalence estimated at ~3.9% [12]. According to the DSM‐5, PTSD may develop following exposure to actual or threatened death, serious injury, or sexual violence and is characterized by four distinct symptom clusters comprising intrusion, avoidance of trauma‐related stimuli, negative alterations in cognitions and mood, and alterations in arousal and reactivity [1]. While PTSD is a formal diagnosis, the term “small‐t trauma” has been used in clinical settings and in the trauma‐related psychotherapies to describe seemingly minor but potentially highly distressing experiences, such as humiliation, rejection, or disappointment, that do not meet the conventional criteria for a traumatic event [13]. Although small‐t trauma is not a formally defined psychiatric disorder, it has a substantial impact on emotional well‐being [14], interpersonal functioning [15], and daily functioning [16]. From a clinical perspective, these impacts highlight the importance of considering early intervention. For example, Korte et al. [17] demonstrated that an early intervention for subclinical trauma showed greater and more rapid symptoms reduction following exposure therapy compared with participants with a full PTSD diagnosis, suggesting that early intervention may also be worth considering for individuals experiencing clinically significant distress following small‐t trauma. In particular, individuals with ASD are more likely to experience adverse events and may develop trauma‐related symptoms even in the absence of a life‐threatening event [18, 19]. A meta‐analysis indicated that the prevalence of bullying victimization among ASD individuals throughout their lives is around 67%, which far exceeds the rate observed in the general population [20, 21]. This experience is not limited to childhood. Research shows that more than half of autistic employees report experiencing bullying or discrimination in the workplace [22]. In addition to frequent exposure to these events, memory‐related characteristics of ASD further contribute to the consolidation of trauma memories. In non‐autistic individuals, memories of adverse events are encoded as episodic memories that are integrated within a broader autobiographical memory network [23]. However, individuals with ASD may be less capable of this integration, partially due to difficulties in emotion regulation and a detail‐focused processing style, resulting in poorly contextualized traumatic memories [24, 25].

In the treatment of trauma‐related disorders, eye movement desensitization and reprocessing (EMDR) is recommended as a first‐line treatment in international clinical guidelines [26, 27]. EMDR, proposed by Francine Shapiro, is a psychotherapy method targeting PTSD or other trauma‐related conditions. During EMDR treatment, the therapist provides alternating bilateral stimulation (BLS) to patients, typically through guided eye movements following the therapist’s moving fingers. Throughout this BLS, EMDR is thought to facilitate the reprocessing of traumatic memories that have not been integrated into the autobiographical memory network [28]. To date, there have been several randomized controlled trials (RCTs) of EMDR for patients with PTSD. The first clinical trial of eye movement desensitization (EMD), which was the prior type of EMDR, was conducted in 1989 involving 22 participants who had experienced traumatic events. After the trial, the participant’s traumatic memories were desensitized, and the effects persisted at a 3‐month follow‐up. Subsequent RCTs have reported the effectiveness of EMDR [29], and it has been reported that EMDR is effective for other psychiatric conditions caused by trauma [30]. Despite the growing evidence for the efficacy of EMDR for both traumatic symptoms and other psychiatric conditions, to date, only a small number of studies have examined EMDR in the ASD population [31]. In addition to EMDR, cognitive behavioral therapy (CBT) is also recommended for trauma‐related symptoms across major international guidelines [26, 27]. While both EMDR and CBT have demonstrated efficacy for PTSD independently, little is known about how a sequential combination of these two approaches may benefit individuals with ASD presenting with small‐t trauma. To our knowledge, no clinical case report has previously described the sequential application of ASD‐adapted EMDR, followed by CBT, for interpersonally‐derived small‐t trauma in an adult with ASD.

This case report describes the clinical course of a sequential EMDR and CBT approach for an adult patient with ASD and small‐t trauma. The therapist initially provided EMDR to reduce trauma‐related symptoms, followed by CBT to address cognitive distortions and current interpersonal difficulties.

## 2. Case Presentation

### 2.1. Design

This clinical case report of combined EMDR and CBT treatment with a single patient who had ASD and small‐t trauma was conducted at Heian‐dori Clinic in Aichi, Japan, between May 2024 and March 2025.

### 2.2. Patient

The patient, referred to here by the pseudonym “Naoko”, was a 25‐year‐old Japanese woman. Some potentially identifying details have been modified or omitted to protect her confidentiality. She was diagnosed with ASD and depression according to DSM‐5 criteria by an experienced psychiatrist (Yutaka Omura). The patient had been seeing the psychiatrist as an outpatient for 3 years and had received pharmacotherapy and occupational therapy. In the clinical setting, the psychiatrist identified some traumatic experiences stemming from interpersonal conflicts, noting that while the threat level was not significant, these experiences negatively affected her social life. Based on these diagnoses and her situation, the psychiatrist referred the patient to the first author, who initiated psychotherapy after several assessment sessions. During the intervention period (May 2024 to March 2025), she received sertraline 75 mg/day, trazodone 75 mg/day plus 25 mg/day as needed for insomnia, and lorazepam 0.75 mg/day. Sertraline had been prescribed at 50 mg/day since January 2022, while trazodone and lorazepam had been prescribed at 25 mg/day and 0.5 mg/day, respectively, since June 2021. During her childhood, she did not have specific developmental difficulties such as delayed speech, but she was highly sensitive to the sounds around her that other children did not notice. Additionally, she was not interested in the popular animated characters that friends or her generation liked; however, she showed curiosity toward the sound of musical instruments and classical music, and she also enjoyed playing the piano. While she did not experience great difficulty with her friends, she was bullied by some close friends who excluded her from a peer group, and she continued to suffer from this bullying after entering junior high school. On the other hand, she made some friends in a brass band club at junior high school, and her interest in music deepened throughout her practice in the club. After she graduated from junior high school, she entered a public high school in the region. In the high school brass band club, she continued to play her musical instrument, but the quality of sound from other members led her to give up on becoming a performer. Her interest in music then shifted from playing the instrument to the theory of music. While she was thinking of majoring in music theory at university after high school graduation, her parents disagreed and recommended that she study a more practical subject. They finally allowed her to study the subject at a public university in Japan and decided not to provide any financial support. Seven years prior to the session, she entered the university and majored in music theory.

### 2.3. Measures

#### 2.3.1. The Impact of Event Scale‐Revised (IES‐R)

The IES‐R is a 22‐item self‐report questionnaire used to assess PTSD symptoms. Each item was rated on a 5‐point Likert scale ranging from 0 to 4, with total scores ranging from 0 to 88. The scale has three subscales, including intrusion, avoidance, and hyperarousal [32].

#### 2.3.2. Patient Health Questionnaire‐9 (PHQ‐9)

The PHQ‐9 was used to evaluate the severity of depressive symptoms. It consists of nine items based on the DSM‐IV criteria for major depressive disorder. Respondents rated the frequency of symptoms over the past 2 weeks on a scale from 0 (not at all) to 3 (nearly every day). Total scores range from 0 to 27, where a score of 10 or higher is generally considered as the cut‐off point for clinically significant depressive symptoms [33].

#### 2.3.3. Overall Anxiety Severity and Impairment Scale (OASIS)

The OASIS was implemented to assess the frequency and intensity of anxiety, as well as its associated functional impairment. This brief 5‐item scale provides a continuous measure of anxiety severity across various anxiety disorders. Each item was scored from 0 to 4, yielding a total score range of 0–20. A score of eight or higher is commonly used to identify individuals with clinically significant anxiety [34].

#### 2.3.4. Overall Depression Severity and Impairment Scale (ODSIS)

The ODSIS was used as a brief measure of depression severity and associated impairment. Similar to the OASIS, this scale comprises five items that evaluate the frequency and intensity of depressive symptoms, along with their impact on daily functioning. Items were rated on a 5‐point scale (0–4), with total scores ranging from 0 to 20. A cut‐off score of eight is typically applied to screen for clinically significant depression [35].

### 2.4. Ethical Considerations and Consent for Publication

This case report describes a single clinical case in routine clinical practice and does not involve any research procedures beyond standard clinical care. Therefore, no formal approval from an institutional review board or an ethics committee was obtained. Written informed consent was obtained from the patient for the publication of the case and its clinical details. Minor textual changes were made to protect the patient’s anonymity without altering the core content.

### 2.5. Intervention

Sessions were conducted every two weeks, with each session lasting for 90 min. From session 19 onward, the CBT follow‐up phase was modified to monthly sessions of 45 min each. Both EMDR and CBT were delivered in accordance with established treatment protocols and the corresponding treatment manuals. The therapist is a certified clinical psychotherapist and had received formal training in both EMDR and CBT. In addition to following the corresponding treatment manuals and protocols, the treatment course was reviewed in regular group‐based clinical case conferences.

### 2.6. Case Formulation

A comprehensive assessment was conducted across multiple sessions, including the collection of developmental history, family history, complaints, and a life chart. Naoko described several positive experiences characterized by personal effort and achievement, including gaining admission to both her first‐choice high school and university and experiencing a sense of achievement through dedicated musical practice. In contrast, interpersonal difficulties were retained as highly distressing memories accompanied by strong emotional responses. Naoko reported that these memories occasionally intruded into conscious awareness without warning, causing her to experience sudden tearfulness and profound fatigue. Among these, two experiences were identified as particularly distressing: (1) an interpersonal conflict within a student organization she had belonged to during university and (2) family‐related conflict.

Naoko further stated, “When something unpleasant happens in front of me, memories from the past suddenly surface, and I become overwhelmed with anxiety.” This suggested that current interpersonal stressors were readily associated with past experiences and that the activation of these memories was likely amplifying both depressive and anxiety symptoms. Additionally, ASD‐related characteristics, including difficulties in interpreting ambiguous social cues and a tendency toward heightened sensitivity to others’ emotional states, were considered to be contributing factors to the intrusive recollections and the worsening depressive and anxiety symptoms. Despite these challenges, Naoko’s academic performance had consistently been above average, and her capacity for persistent, hard‐working effort was identified as a notable personal strength. The full Scale IQ score on the Wechsler Adult Intelligence Scale–Fourth Edition (WAIS), administered 1 year prior to this psychotherapy, was 114. When the therapist asked Naoko to reflect on this capacity for effort, she responded: “When I am feeling well, I am able to work hard in that way. However, when I become depressed or overwhelmed by anxiety, it becomes difficult to look back on my past successes.

Based on these clinical findings, the therapist formulated a case conceptualization in which unprocessed small‐t trauma experiences and ASD‐specific characteristics were hypothesized to interact reciprocally, maintaining a pattern in which past events were automatically retrieved in response to heightened interpersonal stress, thereby perpetuating the patient’s current symptoms.

### 2.7. Course of Treatment

#### 2.7.1. Phase 1: Psychoeducation and Identification of Target Episodes

Prior to beginning EMDR, psychoeducation was provided with the aim of establishing a shared understanding of the treatment rationale and goals. In consideration of Naoko’s ASD characteristics, abstract concepts and explanatory content were simplified to the greatest extent possible, and the therapist continuously checked for comprehension throughout the psychoeducation process. A picture book was used as a visual aid to explain the nature of trauma responses and the mechanisms underlying the processing of distressing memories [36]. Given that partial re‐experiencing and intrusive memory retrieval were anticipated even during the assessment phase, the therapist coordinated with the attending psychiatrist and concurrently worked to develop the patient’s coping skills for managing such states. During this phase, a “safe place” was established. Naoko identified her partner’s room as a space where she felt calm and secure. The container technique was introduced at this stage, and Naoko practiced it during the sessions.

Regarding the identification of target episodes, an exploration of past memories relevant to Naoko’s current presenting complaints and history revealed a chain of interconnected adverse experiences, including early relational difficulties within the family of origin and interpersonal conflicts during her school years. In selecting target memories, the patient noted: “My memories of the distant past are somewhat unclear, whereas more recent events remain vivid.” In response, the therapist decided to initiate intervention with more recently experienced and clearly recalled episodes, for which memory accessibility was relatively higher, and to progress toward earlier and more core traumatic memories once a sufficient sense of safety within the therapeutic relationship had been established.

Two episodes were selected as primary targets, organized as follows. Stage 1 (recent memory): An interpersonal conflict episode in which she experienced feelings of disregard and exclusion by faculty members and students from other departments within a student organization. Stage 2 (core memory): Oppressive and demeaning verbal conduct by her grandmother, spanning childhood through adolescence. Following the processing of these episodes, the therapist planned to collaboratively address additional episodes with Naoko, as appropriate.

#### 2.7.2. Phase 2: EMDR Intervention

Naoko had been responsible for managing and producing a concert involving faculty members and students from the instrumental performance department, making considerable efforts to accommodate each performer’s individual requests. On one occasion, a faculty member who was participating as a performer asked her about her area of study. Upon learning that Naoko was not enrolled in the instrumental performance program, the faculty member responded with a dismissive and condescending remark—“Ah, I thought as much”—which Naoko perceived as highly demeaning. She reported feeling that “My efforts were disregarded, and I was judged and dismissed solely on the basis of my academic specialization.” She felt exhausted by the faculty member’s repeated unreasonable requests. Although Naoko had initially intended to continue her involvement in the student organization, each subsequent encounter with that faculty member and their students intensified her distress, eventually making her continued participation untenable.

During the assessment phase, the most distressing moment (target image) associated with this experience was identified as “the expression on the faculty member’s face as she looked at me coldly.” The Negative Cognition (NC) was established as “I am worthless,” and the Positive Cognition (PC) as “I did my best; I have worth.” Prior to intervention, the Subjective Units of Disturbance (SUD) was rated at 8, and the Validity of Cognition (VOC) score was 2. The associated emotion was sadness, accompanied by a body sensation of generalized physical tension.

At the outset of the desensitization phase, standard eye movements guided by the therapist’s fingers were attempted. However, Naoko reported difficulty managing the dual task of eye tracking and memory retrieval, stating: “It is hard to move my eyes and think at the same time.” Following discussion with Naoko, the BLS modality was changed to the butterfly hug technique. During reprocessing, Naoko initially described the situation in detail. Gradually, statements reflecting a temporal differentiation between past and present began to emerge, such as: “I probably won’t have any further involvement with that person.” Further cognitive shifts followed, including: “Living in the world of production and performing arts is not suited to me,” “That world is not for me—but I am glad I realized that early on,” and “Being devoted to one’s craft is the work of a performer.” These shifts reflected a transformation in Naoko’s appraisal of the original memory—from a source of pain to a significant turning point in her life trajectory.

Across a total of three sessions addressing this episode, the SUD decreased from 8 to 4; however, residual physical tension remained, and reprocessing was continued. Naoko was able to express—through imagery—what she had been too frightened to say directly at the time: “Isn’t that kind of attitude a problem for someone in a position of authority?” and “Because you act so arrogantly, the students follow suit.” Following this, the SUD decreased further, and the physical tension gradually resolved. Upon completion of the fourth reprocessing session, the final SUD was 2, and the VOC for the PC “I did my best; I have worth” had increased to 7. Reprocessing of the student organization memory was thereby concluded.

During the reprocessing of the student organization episode, Naoko had spontaneously recalled interactions with her grandmother as a formative experience of powerlessness in the presence of authority figures. She reported that, during her second year of middle school, her grandmother had made remarks such as, “You’re a girl, so you should do as your parents say,” and “I wonder whether it’s really necessary for a girl to pursue higher education.” These comments constituted direct criticism based solely on Naoko’s gender. The NC for this episode was established as “I am powerless” and the PC as “I am fine just as I am.” Prior to intervention, the SUD was 8 and the VOC was 1. The associated emotions were anger and helplessness, accompanied by a physical sensation of constriction in the throat. Reprocessing using the butterfly hug technique was initiated, intense anger emerged as the patient repeatedly articulated the injustice of what she had experienced. She expressed particular anguish over her parent’s agreement with the grandmother’s remarks. Gradually, she began to reframe her perception, reaching cognitive shift such as: “My grandmother was a person bound by outdated values,” and “My grandmother’s remarks reflect her own limitations—they have nothing to do with my worth.” These insights were accompanied by the complete resolution of the physical distress associated with the episode. The final SUD reached 0, and the VOC for the PC “I am fine just as I am” increased to 7.

#### 2.7.3. Phase 3: CBT Intervention

Following the completion of EMDR, Naoko reported that while past events occasionally came to mind, they were rarely accompanied by significant psychological distress, and she had successfully commenced employment. However, she noted experiencing marked tension when interacting with a supervisor whose interpersonal style she found particularly challenging. Naoko observed that prior to EMDR, such interpersonal stressors would have immediately triggered associations with past traumatic memories and produced trauma‐like symptoms; crucially, this pattern was no longer present. In response to her feeling uncertain about how to conduct herself in these situations and holding the automatic thought, “She must dislike me,” the therapist proposed a CBT‐based follow‐up, and Naoko agreed.

Each subsequent session focused on specific encounters with this supervisor using cognitive restructuring techniques. She reported an incident where she greeted the supervisor but received no response, resulting in sadness, mild anxiety, and automatic thoughts such as: “I am not performing well at work,” “I am not being evaluated positively,” and “My colleagues must also think poorly of my work.” Over the course of subsequent CBT sessions, she accumulated corrective experiences, including being entrusted with an increasing range of responsibilities, developing positive relationships with peers and senior colleagues in her department, and learning that other colleagues also found the supervisor difficult. Through this process, Naoko adopted the adaptive perspective: “In any setting, there will always be people I find difficult to get along with.” Consequently, she reported a reduction in psychological distress during interactions with the supervisor.

## 3. Results

The outcome measures demonstrated clinically meaningful reductions across traumatic symptoms, depressive, and anxiety symptoms through the course of treatment. As shown in Table 1, scores on all measurements improved from pretreatment to post‐EMDR, and these results were maintained during the subsequent CBT follow‐up. Following EMDR treatment, Naoko secured employment at a company under a disability employment framework and has remained employed to date. Additional psychosocial improvements were also noted across multiple domains. These included the improvement of romantic relationship, enhanced relationships with coworkers and family members, and improved capacity for self‐care.

**Table 1:** Changes in clinical outcome measures across treatment phases.

Variable	Pre	Post‐EMDR	Post‐CBT
IES‐R	56	18	8
PHQ‐9	16	8	5
OASIS	15	9	5
ODSIS	15	10	3

Abbreviations: IES‐R, Impact of Event Scale‐Revised; OASIS, Overall Anxiety Severity and Impairment Scale; ODSIS, Overall Depression Severity and Impairment Scale; PHQ‐9, Patient Health Questionnaire‐9.

## 4. Discussion

This clinical case report describes clinical improvement following a sequential EMDR and CBT approach in an adult patient with ASD and small‐t trauma. In general, adverse life events that do not qualify as Criterion A traumatic events tend to be underrecognized as targets for psychiatric treatment. However, a close and complex relationship exists between neurodevelopmental disorders and trauma [37]. Due to sensory hypersensitivity, atypical cognitive processing styles, and qualitative impairments in social interaction, individuals with ASD are likely to experience events that neurotypical individuals might process as transient discomfort as intensely frightening or humiliating, retaining vivid memories of such experiences over extended periods [37–39]. In the present case, an accumulation of interpersonal stressors and psychologically overwhelming interactions within the family of origin contributed to the consolidation of negative cognitions such as “I am worthless” and “I am powerless.” These experiences gave rise to flashback‐like intrusive recollections and chronic hyperarousal, ultimately impairing social functioning in domains including employment and interpersonal relationships.

The interventions in this case report have several clinical strengths. First, we modified some points of the standard EMDR protocol. The standard EMDR protocol recommends eye movements as the primary form of BLS. However, it has been noted that in individuals with ASD, eye movements carry the risk of inducing sensory distress [40]. In the present case, the patient experienced difficulty simultaneously managing two concurrent demands: retrieving traumatic memories while visually tracking the therapist’s moving fingers. Experimental research has demonstrated that eye movements impose a greater load on working memory than other forms of BLS [41]. The prompt transition from eye movement to the butterfly hug technique may have reduced excessive working memory load and facilitated memory reprocessing. Indeed, following the switch from eye movements to the butterfly hug technique as BLS, the patient was able to recall memories more easily and respond more quickly to the therapist’s questions. Second, the “container technique” was introduced during the assessment phase, in which the patient engaged in guided imagery to symbolically set aside memories and emotions that could not be fully processed within the session. This is considered a factor in reducing treatment‐related anxiety and enabling treatment completion. Finally, initially targeting a recent memory rather than an older one may also have been beneficial in this case. The standard EMDR protocol indicates that treatment should begin with the earliest touchstone memory that underlies the current symptomatology [28]. In the present case, this corresponded to experiences within the family context; however, the patient demonstrated avoidance and fear associated with these memories, and the memories themselves lacked sufficient clarity. By starting with memories that were easier for the patient to recall, the therapist may have helped the patient experience a reduction in SUD during treatment, which may have increased her motivation for treatment.

Furthermore, the sequential delivery of EMDR for trauma processing, followed by CBT, could be a consolidation intervention for adults with ASD experiencing trauma‐related symptoms. In cases of ASD presenting with trauma responses, attempting verbal cognitive restructuring during periods of frequent hyperarousal and flashbacks may yield limited learning outcomes due to functional neurological constraints [31]. In the present case, EMDR first appeared to reduce the subjective trauma‐related hyperarousal associated with past traumatic events, enabling the patient to differentiate experientially between “the past” and “the present” and to achieve a meaningful reduction in chronic fear. Subsequently, the introduction of CBT allowed the patient, in collaboration with the therapist, to observe her own cognitive patterns more objectively in relation to present‐day difficulties, ultimately facilitating adaptive responses to real‐world challenges.

While clinical research on the treatment of trauma‐related disorders has been conducted, few studies have focused on patients with ASD or on small‐t trauma in this population. Given the perceptual characteristics of individuals with ASD, standardized treatment protocols may not always be directly applied to this population. This case report suggests that considering the clinical characteristics of individuals with ASD may be necessary while highlighting the potential benefit of modifying therapeutic techniques and combining EMDR with CBT.

### 4.1. Limitations and Future Directions

This case report has several limitations. First, this is a single clinical case report. The findings cannot be generalized to other patients with ASD. Second, since the patient received concomitant treatment, including pharmacological therapy, the observed reduction in symptoms cannot be solely attributed to the psychotherapy intervention. According to the international clinical guidelines for PTSD, selective serotonin reuptake inhibitors (SSRIs) such as sertraline are recommended as the first‐line medication for PTSD and may be used in conjunction with psychotherapy for trauma‐related symptoms [42]. Pharmacotherapy has been associated with reductions in depressive and anxiety symptoms in trauma‐exposed populations [43, 44]. Thus, medication‐related effects represent an important potential confounder in interpreting the clinical improvement observed in this case. Third, individual treatment sessions were not recorded, and no formal measures of treatment fidelity, such as adherence checklists or independent fidelity ratings, were implemented. Therefore, future research should examine integrated or sequential EMDR and CBT for adults with ASD and small‐t trauma using more rigorous designs.

## 5. Conclusion

Despite these limitations, this case report provides meaningful clinical insights into the psychotherapeutic approach for adults with ASD who have experienced small‐t trauma. While EMDR and CBT may be effective individually, this case suggests that a sequential EMDR and CBT approach may help address trauma‐related symptoms while accommodating ASD‐related characteristics, such as sensory sensitivity, meaningful clinical insights, and difficulties in processing ambiguous interpersonal cues. Although further research is required, the present case provides a clinical illustration of how EMDR adapted to the characteristics of ASD, followed by CBT, may be used to address trauma‐related symptoms associated with small‐t trauma in adults with ASD.

## Author Contributions

Keita Kawai conceived the study design, conducted all psychotherapy sessions including EMDR and CBT interventions, performed the clinical assessment, reviewed the relevant literature, and drafted the manuscript. Michie Ishida contributed to the clinical assessment of the participant and critically reviewed and revised the manuscript. Yutaka Omura provided psychiatric diagnosis, initiated and managed pharmacological treatment, referred the participant to psychotherapy, and critically reviewed the manuscript.

## Funding

This research was supported by the Canon Foundation in Europe research fellowship grants (to Keita Kawai). Open Access funding was enabled and organized by Projekt DEAL.

## Disclosure

All authors approved the final version of the manuscript for submission.

## Conflicts of Interest

The authors declare no conflicts of interest.

## Data Availability

The clinical data supporting this case report are not publicly available because they contain information that could compromise patient confidentiality.
